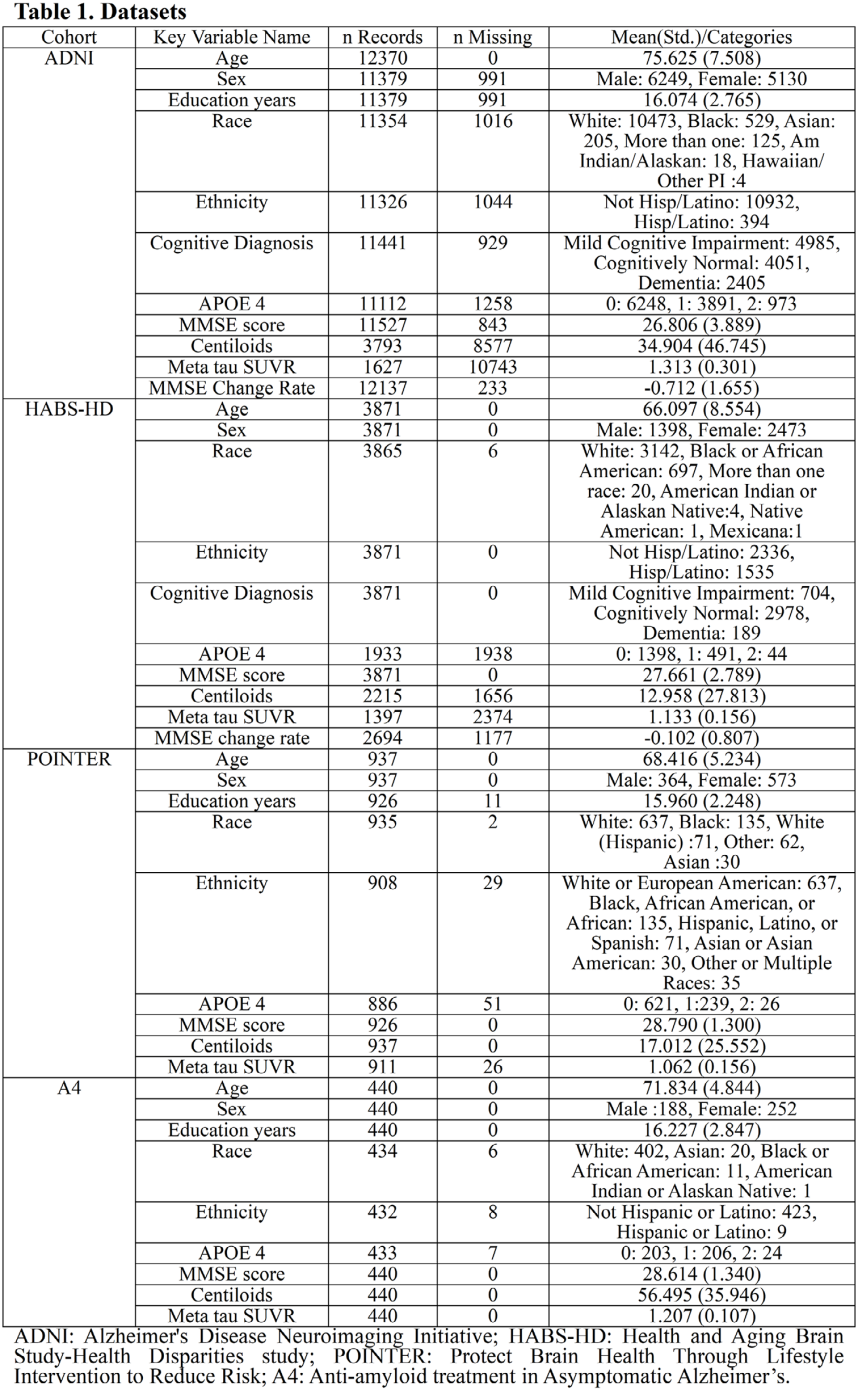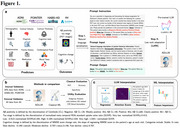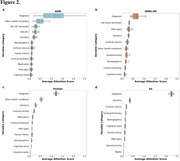# Enhanced language models for predicting Alzheimer's disease pathology

**DOI:** 10.1002/alz70860_105841

**Published:** 2025-12-23

**Authors:** Qiuran Rita Lyu, Yanping Li, Kevin Shen, Marcell Borhi, Susan M. Landau, William J. Jagust, Joseph Giorgio, Jingshen Wang

**Affiliations:** ^1^ University of California, Berkeley, Berkeley, CA, USA; ^2^ Nankai University, Tianjin, Tianjin, China; ^3^ The Nueva School, San Mateo, CA, USA; ^4^ Indiana University Bloomington, Bloomington, IN, USA; ^5^ Neuroscience Department, University of California, Berkeley, Berkeley, CA, USA; ^6^ Department of Epidemiology, School of Public Health, University of California, Berkeley, CA, USA; ^7^ Lawrence Berkeley National Laboratory, Berkeley, CA, USA; ^8^ University of California, San Francisco, San Francisco, CA, USA

## Abstract

**Background:**

The ability to predict individual‐level biomarker characteristics, along with cognitive decline, could represent a major advance in the care of AD patients. Advanced language models (LLMs), with their ability to interpret natural language descriptions of structured data, offer a powerful technique for uncovering latent relationships and integrating diverse predictors to make interpretable predictions about AD.

**Method:**

We trained ADLLM, an enhanced LLM based on LLaMA 3.1, fine‐tuned using Low‐Rank Adaptation (LoRA) on serialized data from three cohorts: ADNI, HABS‐HD, and POINTER, using A4 for external validation (Table 1). Predictors included demographics, neuroimaging and fluid biomarkers, clinical assessments, etc. Outcomes were Aβ and tau burden in all cohorts, and rate of MMSE change in ADNI and HABS‐HD. Inputs were converted to a text prompt (Figure 1a), allowing the model to capture relationships across modalities and predict composite outcomes. Performance was evaluated using multi‐class AUC, accuracy, and specificity (Figure 1b), based on the predicted probabilities generated by ADLLM, with the predicted category assigned to the one with the highest probability. We used multiple methods for LLM interpretation quantifying the importance of variable groups across cohorts (Figure 1c).

**Result:**

ADLLM demonstrated strong hold‐out predictive performance across the training cohorts. For Aβ burden prediction, it attained AUCs of 0.825 (ADNI), 0.773 (HABS‐HD), and 0.702 (POINTER). For tau burden prediction, AUCs were 0.730 (ADNI), 0.597 (HABS‐HD), and 0.516 (POINTER). For MMSE change rate prediction, the model achieved AUCs of 0.806 (ADNI) and 0.816 (HABS‐HD). Attention score analysis revealed cohort‐specific variable importance for predicting three outcomes simultaneously, with AD cerebrospinal fluid biomarkers playing a dominant role in ADNI and plasma biomarkers being critical in HABS‐HD (Figure 2a–c). In external validation with the A4 dataset, ADLLM maintained generalizability, achieving AUCs of 0.610 for Aβ burden and 0.600 for tau burden.

**Conclusion:**

ADLLM can predict composite outcomes by processing multimodal inputs, capturing longitudinal patterns, and providing interpretable attention‐based variable importance. The model consistently performed well across cohorts and outcome tasks, emphasizing its potential as a scalable and clinically relevant tool for AD diagnosis and progression monitoring.